# Biofeedback rehabilitation in patients with binocular inhibition due to macular disease

**DOI:** 10.1007/s00417-025-06749-1

**Published:** 2025-01-25

**Authors:** Valeria Silvestri, Paola Piscopo, Simona Turco, Filippo Amore, Stanislao Rizzo, Mark S. Mandelcorn, Luminita Tarita-Nistor

**Affiliations:** 1 National Center of Services and Research for the Prevention of Blindness and Rehabilitation of the Visually Impaired, Fondazione Policlinico Universitario Agostino Gemelli, IRCCS, Largo Agostino Gemelli N°8, 00168 Rome, Italy; 2 UOC Oculistica, Fondazione Policlinico Universitario Agostino Gemelli-IRCCS, Rome, Italy; 3 Donald K Johnson Eye Institute, Krembil Research Institute, University Health Network, Toronto, Canada; 4 Department of Ophthalmology and Vision Sciences, University of Toronto, Toronto, Canada

**Keywords:** Visual impairment, Central vision loss, Biofeedback, Binocular inhibition, Microperimetry

## Abstract

**Background:**

To investigate whether patients with binocular reading inhibition due to central vision loss benefit from a new biofeedback (BF) rehabilitation method that aimed at improving fixation stability and at establishing a correspondence between the monocular preferred retinal loci (PRLs) on functioning retina in both eyes.

**Methods:**

Thirty-three patients with bilateral macular disease and with binocular reading inhibition participated in 10 training sessions consisting of 10-min visual stimulation for each eye to stabilize fixation and relocate the PRL (if needed) using the BF module of the MP-1 microperimeter (Nidek Technologies Srl., Vigonza, PD, Italy). Binocular and monocular reading performance, contrast sensitivity, and visual acuity were evaluated pre and post training. Binocular summation/inhibition was evaluated with binocular ratio (BR).

**Results:**

Fixation stability improved significantly post training in both eyes. Maximum reading speed during binocular viewing increased from 57 ± 24wpm pre training to 67 ± 24wpm post training. BR increased for all parameters of reading, visual acuity, and contrast sensitivity. Training resulted in a complete reversal of binocular reading inhibition in 30% of patients.

**Conclusions:**

For patients with binocular inhibition due to central vision loss, BF training to stabilize fixation and to bring the monocular PRLs into correspondence on functioning retina in both eyes is an efficient rehabilitation method to improve binocular performance.

## Abstract (highlights)

**Key messages:**

*****What is known***:**

- Some patients with macular disease may suffer from binocular inhibition, when binocular performances are worse than the monocular performances with the better eye.
- Binocular inhibition represents an additional impairment to the already reduced visual acuity, contrast sensitivity, fixation stability and reading performance.

*****What is new***:**

- A new biofeedback training method was used to improve fixation stability in both eyes and to bring the monocular preferred retinal loci into retinal correspondence.
- This biofeedback rehabilitation procedure resulted in an increase in binocular reading speed and in a relief of binocular reading inhibition.

## Introduction

Macular diseases produce central retinal lesions that are often unequal in the two eyes. Aside from the loss of central vision and disturbances in the oculomotor control, binocular visual functions such as stereopsis and binocular summation are compromised [1–8].

Binocular summation occurs when binocular performance is superior to monocular performance on various visual tasks and is a typical function of the healthy visual system. Good eye alignment and equal monocular foveal inputs are requirements for an efficient binocular summation mechanism. Binocular performance can be worse than the monocular performance with the better eye in patients with central vision loss [1–5], especially in those with unequal damage in the two eyes, despite their generally good eye alignment [8–10]. This is known as binocular inhibition and represents an additional layer of impairment to those that are already generated by the loss of the central vision in each eye.

An inadequate oculomotor adaptation to the loss of central vision may be an important factor that can cause binocular inhibition [4, 5]. When central vision is affected, the fovea is damaged and this results not only in impairments in performing high resolution tasks, but also in the loss of the reference point of the oculomotor system. A functioning adaptation to the loss of the fovea is the consistent use of a part of the eccentric retina to perform visual tasks; this pseudo-fovea is called the preferred retinal locus (PRL) and its precise location on the retina can be identified during a fixation test with imaging instruments such as microperimeters [10]. Unfortunately, these instruments permit only monocular recordings, and the literature has revealed that the monocular PRLs of the two eyes often are not in corresponding retinal locations [9–11]. Direct and indirect evidence from recordings with eye-trackers show that oculomotor control is typically driven by the better eye [12–15]. This implies that during binocular viewing, the PRL of the worse eye must come in corresponding retinal location with that of the better eye, even at the expense of it falling onto scotoma [12]. Therefore, in such cases, the visual inputs from the two eyes are severely imbalanced and this can explain the binocular inhibition. Indeed, it has been shown that many patients with macular disease suffer from binocular inhibition of visual acuity, contrast sensitivity, and reading performance [1–5], and that those with monocular PRLs in non-corresponding locations are more likely to show this impairment [4, 5].

For example, our recent work on a large sample of patients with macular disease showed that about 40% of them experienced binocular reading inhibition [5]. We found that these individuals had the lowest binocular reading speed and generally had monocular PRLs in non-corresponding locations: the PRL in the better eye located temporal or nasal to the central lesions. Moreover, subjects with binocular inhibition presented the largest interocular acuity difference and lacked residual stereopsis. To date, there is no effective medical intervention to alleviate binocular inhibition in these patients.

In recent decades, the biofeedback (BF) training module incorporated in microperimeters such as the Nidek MP series (i.e., MP1, MP3; Nidek Technologies Srl., Vigonza, PD, Italy) and CenterVue Macular Integrity Assessment (MAIA; CenterVue, Padua, PD, Italy) has been used with relative success to improve visual function in patients with macular disease [16]. Using various schedules, BF training combines acoustic and visual inputs to guide better fixational oculomotor control and to direct the development of a new PRL at a more advantageous retinal location, with the general goal to stabilize fixation and to relocate the PRL to a position that would allow for a larger visual span necessary for better reading performance [17–20]. It is not yet known if this rehabilitation technique can be used to improve binocular function in patients with binocular inhibition. Due to technical limitations of these devices, only monocular training is possible and, ideally, a rehabilitation method should aim at improving fixation and relocating the PRLs to corresponding locations on functioning retinas in both eyes. Recently, it has been suggested that the PRL – and therefore its relocation – should be considered in the context of binocular viewing, and that a particular attention should be given to whether the examined PRL is in the better or in the worse eye, because their properties can differ vastly [9, 10]. For example, if the training is designed only for the PRL in the worse eye without any considerations for the PRL in the better eye, the rehabilitation may result in no benefit at all, since the properties of this trained PRL can change during binocular viewing to come into correspondence with the untrained PRL in the better eye.

The purpose of this study was to investigate the effect of a new use of the BF rehabilitation technique, designed to improve the oculomotor control in both eyes and to bring the monocular PRLs in corresponding locations (if required) in patients with binocular reading inhibition due to central vision loss. Maximum reading speed was used as a criterion to determine binocular inhibition. We examined whether the oculomotor changes achieved through BF training translated into better visual binocular functions on various tasks and in improvements of the binocular summation mechanism. Given the severity of vision impairment in these patients, broader rehabilitation methods are warranted.

## Methods

### Study design and participants

For this prospective, case series study, 33 (15 male and 18 female) participants with a mean age of 69 ± 12 years were recruited among those who access the National Centre of Services and Research for the Prevention of Blindness and Rehabilitation of the Visually Impaired, International Agency for the Prevention of Blindness-IAPB Italy, sited in Fondazione Policlinico Agostino Gemelli IRCCS, Rome, Italy. The eligibility criteria were a minimum age of 18 and maximum age of 80 years, a diagnosis of central vision loss due to macular diseases, a best corrected visual acuity (BCVA) between 0.5 LogMAR and 1.0 LogMAR in the better eye, unstable or relatively unstable fixation on fixation microperimetric examination, and a stable visual impairment from at least 3 months. Among these patients, 25 suffered from nonexudative age-related macular degeneration, 7 were diagnosed with Stargardt disease, and 1 had cone-rod dystrophy. All patients spoke Italian language fluently and no one had cognitive impairment. Patients were excluded if they had other concomitant eye diseases, significant media opacities and if they performed ocular surgery within the previous 90 days. The study was approved by the Ethical Committee of the Fondazione Policlinico Universitario Agostino Gemelli IRCCS (Rome, Italy) and conducted in accordance with the tenets of the Declaration of Helsinki. A written informed consent was obtained from all the patients included in this study. All 33 patients were able to complete the rehabilitation program.

### Intervention

All subjects underwent a complete ophthalmic evaluation at baseline and after BF stimulation, as follows. BCVA was measured monocularly and binocularly using the Early Treatment of Diabetic Treatment Study (ETDRS) charts at 4 m (Precision Vision, Bloomington, IL) in LogMAR notation. Reading acuity, critical print size and maximum reading speed were evaluated with the Italian version of the MNRead charts (www.precision-vision.com) at 25 cm with an addition of + 4 spheric lens (1 x) to the distance refractive correction monocularly with the better eye and binocularly. Monocular and binocular contrast sensitivity (CS) was evaluated with the Pelli-Robson chart (www.precision-vision.com) at 1 m adding + 1 spheric lens. The Stereo Fly Test (PrecisionVision, Woodstock, IL, US) was used to measure stereo acuity and was administered according to manufacturer’s instructions. The experimenter held the test straight in front of the patient to maintain the proper axis of polarization at 40 cm and to avoid light reflections on the shiny surfaces. During the test, patients were asked to wear the optical correction for the test distance under the test’s polarized viewers. In addition, a slit-lamp examination and binocular ophthalmoscopy were performed. Microperimetric retinal sensitivity, color fundus photography, PRL location, and fixation stability were obtained for each eye using the MP-1 microperimeter (Nidek Technologies Srl., Vigonza, PD, Italy) after pupil dilation. During the fixation exam, the patients were instructed to fixate on a 2 deg white cross projected on a black background for 15 s, with their head stabilized on the headrest of the instrument. For patients with poor visual acuity the cross was increased to 3 deg. The background luminance of the MP-1 microperimeter was 1.27 cd/m^2^ (= 2 abs). At the end of the fixation examination, a fundus photograph was obtained. The better- and the worse-seeing eyes were identified as the eyes with the better and the worse visual acuity, respectively.

### MP-1 biofeedback training

Visual stimulation was performed using the BF training module of the MP-1. Training was done monocularly for each eye and consisted of instructions to move the eye such as the gaze reached a target (i.e., a white cross) projected in a specific retinal location selected by the experimenter, who was a certified orthoptist (author VS). When the eye moved towards this location, the BF module emitted an audio signal whose frequency intensified as the gaze approached the chosen target. The signal became continuous when the gaze reached the target and became discontinuous when the gaze shifted far from the selected area. Once the patients were fixating in the correct position and the audio signal was continuous, they were asked to maintain the gaze position at that location for as long as possible. Meanwhile, a reversal black and white checkboard pattern was automatically superimposed onto fixation target. The reversal checkerboard’s size was selected according to the patient’s visual acuity and represented a flickering pattern with high spatial frequency and low temporal frequency. The reversal checkboard had a retina coverage of 8 deg and single elements of 0.5 deg. For patients with a visual acuity between 0.9 and 1 LogMAR the single elements of the checkboard were set at 1 deg. The flickering was set to a temporal frequency of 5 Hz. The flickering pattern stimulus was used because it has been shown to be more effective for fixation stability, reading speed and retinal sensitivity when compared with only auditory biofeedback [21–23].

Before starting with the BF training, patients were instructed by the same certified orthoptist about how to fixate and move their eyes according to the audio signal. BF training was performed in both eyes. The goals of the training were: 1) to improve fixation stability in both eyes, 2) to establish a correspondence between the two PRLs on functioning retina in both eyes for those who did not have this characteristic at baseline, and 3) in cases that allowed it, to relocate the PRLs in locations that are more suitable for reading (i.e., locations that allow for larger visual span). The training sessions were scheduled twice a week for a total of 10 BF sessions (5 weeks). Each BF session ran 10 min long per eye. No mydriatic eye drops were instilled during the training phase.

### Outcome measures

The pre and post BF training outcome measures were maximum reading speed as well as critical print size, reading acuity, visual acuity, and contrast sensitivity during binocular and monocular viewing with the better eye. In addition, PRL location was defined according to Fuji classification [24]; for the PRLs classified as eccentric, the location was specified as temporal, nasal, superior, or inferior retinal locations. The radial grid embedded in the MP-1 was used to measure the PRL distance from the former fovea and to evaluate scotoma size. Fixation stability was evaluated at the initial PRL location and at the trained location at the end of the training.

### Data analysis

Fixation stability was evaluated with the bivariate contour ellipse area (BCEA) encompassing one standard deviation (1 sd, 68.2%), which was provided in the fixation test output of the MP-1 and measured in deg^2^. Because of concerns about spurious results and relatively high test–retest variability of fixation stability [25], two consecutive fixation examinations were performed for each eye pre and post training. Paired-samples t-tests showed that there was no significant difference in BCEA and PRL distance from the former fovea for the two consecutive examinations, and therefore we report their average. The scotoma was defined as the central lesion where the patient did not recognize the brightest stimuli in decibels (absolute scotoma). Its size was measured manually by overlapping the polar grid to the microperimetric exam and delineating the diameter in horizontal and vertical direction up to the border at which the stimuli were not seen. The scotoma size was reported as the average of the horizontal and vertical measurements.

Binocular summation or inhibition was determined with binocular ratio (BR) such as a BR value greater than 1.05 would indicate summation, smaller than 0.95 would indicate inhibition, and between 0.95 to 1.05 would indicate equality [3, 6]. To abide by this rule, the calculation of this ratio depends on the unit of measurement to express binocular superiority. As a result, BR was calculated as the ratio between binocular to monocular performance with the better eye for the maximum reading speed because a higher reading speed value indicates better performance, and as the ratio between monocular to binocular performance for the reading acuity, critical print size, visual acuity, and contrast sensitivity because a smaller numerical value indicates superior performance. Data were analyzed with paired-samples t-tests, independent-samples t-tests, and Pearson moment product correlations, using an alpha level smaller than 0.05.

## Results

### Effect of biofeedback training on fixation stability and PRL location

**Fig. 1:**
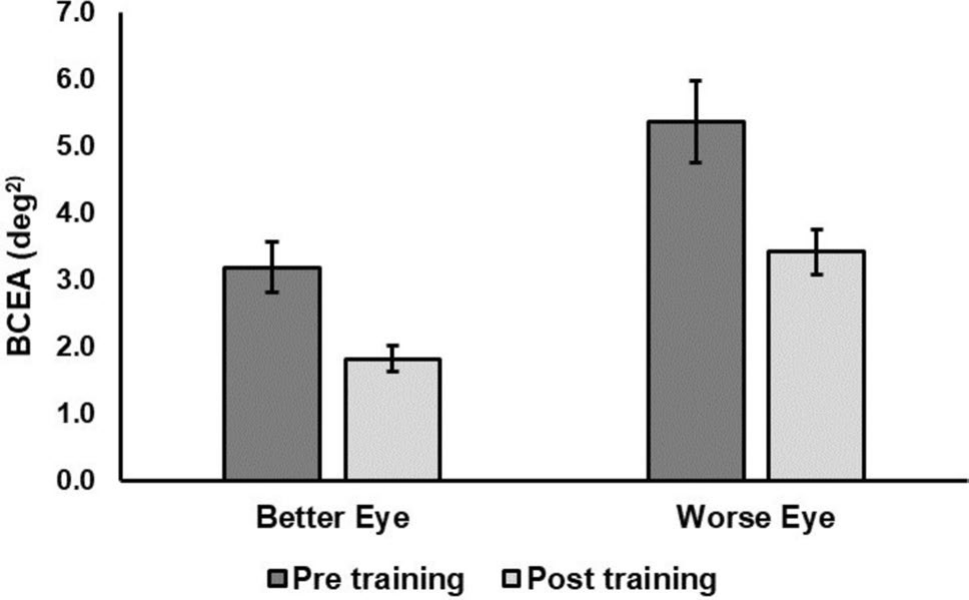
Fixation stability for the better and the worse eye, pre and post training. Error bars represent ± 1SE

For the better eye, fixation stability improved significantly after training from 3.19 ± 2.14 deg^2^ to 1.82 ± 1.17 deg^2^, t(32) = 4.71, *p* < 0.001, but PRL distance from the former fovea changed negligibly from 5.00 ± 2.28 deg to 4.75 ± 2.21 deg. Likewise, for the worse eye, training resulted in a significant improvement in fixation stability from 5.37 ± 3.49 deg^2^ to 3.42 ± 1.93 deg^2^, t(32) = 4.35, *p* < 0.001, and a small but significant change in PRL distance from the former fovea from 6.82 ± 2.23 deg to 6.33 ± 2.28 deg, t(32) = 3.9, *p* < 0.001. Fixation stability results are shown in Fig. 1.

Interestingly, the improvement in fixation stability depended on the initial performance, in the sense that the gain in this measure post training (measured as the difference in fixation stability between pre and post training) was more pronounced for patients with poorer pre training fixation stability. There was a significant positive correlation between pre training fixation stability and the gain in this measure post training for the better eye, r(31) = 0.84, *p* < 0.001, and for the worse eye, r(31) = 0.81, *p* < 0.001.

**Fig. 2:**
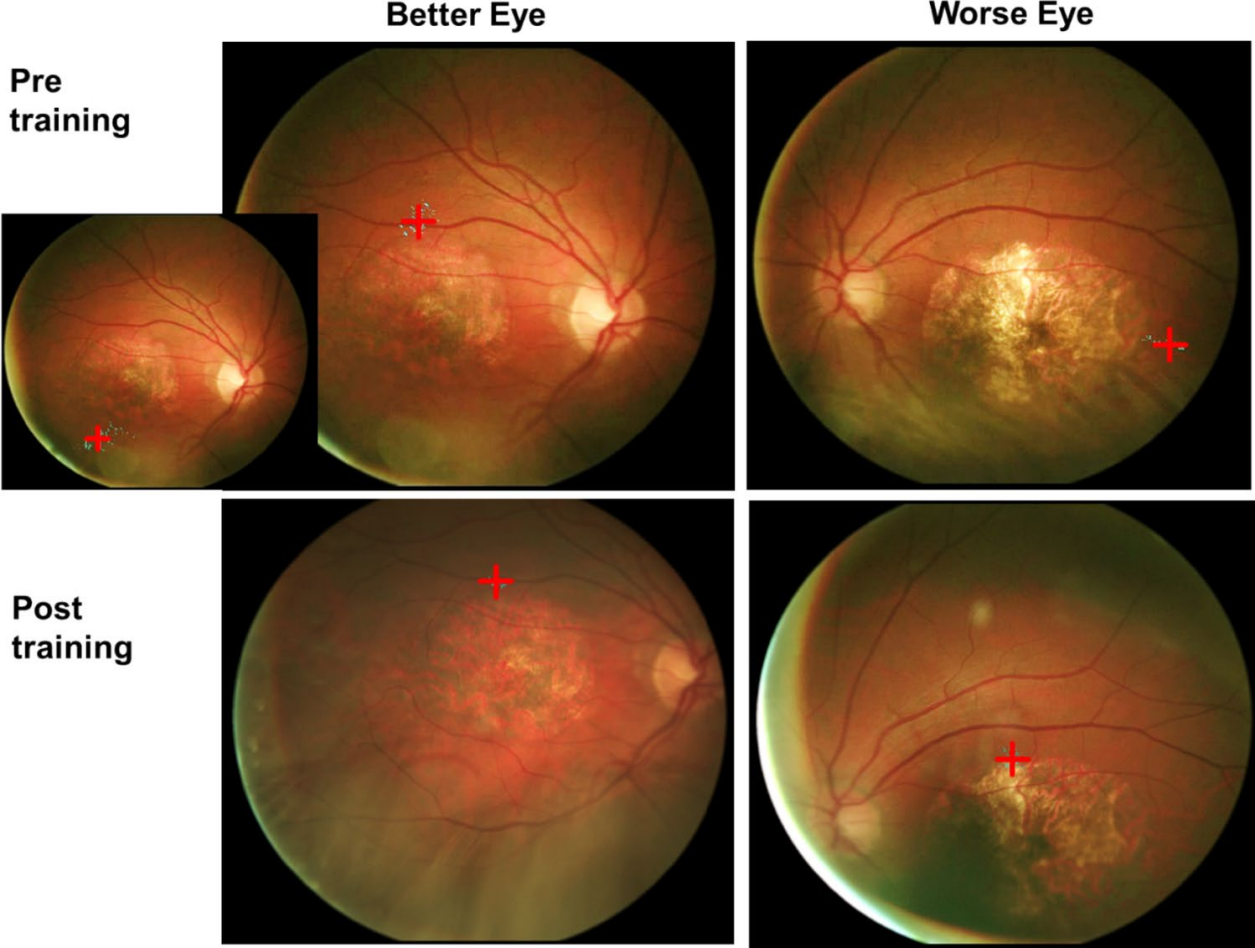
Example of a patient with poor fixation stability and PRLs in non-corresponding locations pre training. The patient also had 2 PRLs in the better eye, one above and one below the lesion (top left insertion); the PRL above the lesion was trained. Fixation stability improved from 3.65 deg2 to 0.4 deg2 in the better eye and from 6.4 deg2 to 2.25 deg2 in the worse eye, and the PRLs moved in corresponding locations post training. Visual acuity was 0.64 LogMAR in the better eye and 0.88 LogMAR in the worse eye before training. After training, a slight improvement was found in both the better eye (0.60 LogMAR) and in the worse eye (0.78 LogMAR)

Pre training, the PRLs in the two eyes were in corresponding locations in 17 cases (52%), and in non-corresponding locations in 16 cases (48%). Post training, 24 patients had the PRLs in corresponding location (i.e., 73%), and only 9 patients had them in non-corresponding locations (i.e., 27%). Figure 2 presents the example of a patient with large lesions in both eyes who had poor fixation stability and PRLs in non-corresponding locations pre training. The patient showed a remarkable improvement in fixation stability (i.e., from 3.65 deg^2^ to 0.4 deg^2^ in the better eye and from 6.4 deg^2^ to 2.25 deg^2^ in the worse eye), and the PRLs moved in corresponding locations post training. This was the only patient in our sample with two PRLs in the better eye (one above the lesion and a more unstable one below the lesion); the PRL above the lesion was trained.

### Maximum reading speed and binocular ratio

**Fig. 3:**
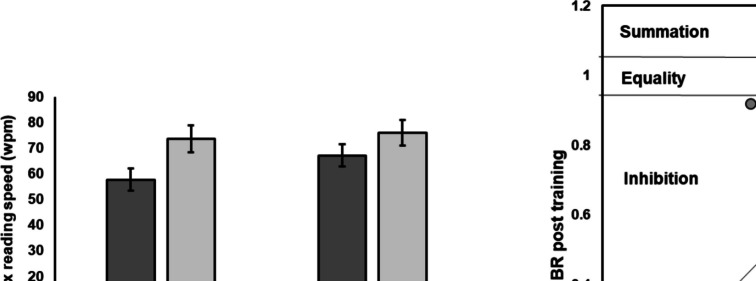
Maximum reading speed during binocular and monocular viewing with the better eye (left panel) and binocular ratio (BR) for maximum reading speed (right panel) pre and post training. Error bars are ± 1 SE

Maximum reading speed during binocular viewing increased significantly from 57 ± 24 wpm pre training to 67 ± 24 wpm post training, t (32) = 6.3, *p* < 0.001. Moreover, there was a statistically significant increase in the reading speed during monocular viewing with the better eye from 73 ± 30 wpm to 76 ± 29 wpm, t (32) = 2.6, *p* = 0.013, but the marginal 3 wpm improvement is within the test–retest repeatability limits of the MNRead test (which is ± 8.6 wpm for central vision loss) [26]. BR increased significantly from 0.78 ± 0.12 pre training to 0.89 ± 0.10 post training, t (32) = 4.8, *p* < 0.001. Maximum reading speed and BRs pre and post training are shown in Fig. 3.

### Subgroup of patients with resolved binocular inhibition

For 10 out of 33 patients (i.e., 30%) training resulted in a reversal of binocular inhibition. For them, BR improved from 0.76 ± 0.16 pre training (i.e., binocular inhibition) to 1.00 ± 0.05 post training (i.e., binocular equality). These patients experienced a larger increase in maximum reading speed during binocular viewing from 52 ± 23 wpm to 69 ± 17 wpm, while the improvements in performance with the better eye remained clinically marginal (i.e., only 3 wpm, from 67 ± 19 wpm to 70 ± 20 wpm). Among these, 7 patients had their PRLs in non-corresponding locations pre training, but this number was reduced to only 2 post training.

### Improvements in binocular function evaluated with other clinical measures: stereoacuity, visual acuity, contrast sensitivity, reading acuity, and critical print size

Although maximum reading speed was used as the criterion for selecting the patients with binocular inhibition in this study, we examined whether training also resulted in binocular improvements in stereoacuity, visual acuity, contrast sensitivity, reading acuity, and critical print size. Stereoacuity pre and post training remained unchanged, with a mean of 2322 ± 1661arcsec. Residual stereoacuity was found in 58% of cases pre training, and this proportion did not change post training. For all the other measures (i.e., visual acuity, contrast sensitivity, reading acuity, and critical print size), binocular and monocular performance with the better eye improved significantly post training. Also, BR of all measures (except for that of contrast sensitivity with marginal significance) had a significant improvement post training. The largest gain was in critical print size, with improvements of 0.13 logMAR in binocular performance and 0.08 in BR. The results are presented in Table 1. The patients had stable disease, with scotoma size pre and post training of 12.8 ± 2.4 deg for the better eye and 14.7 ± 2.8 deg for the worse eye.

**Table 1:** Mean (± standard deviation) for binocular and monocular performance with the better eye, along with binocular ratio (BR) for visual acuity, contrast sensitivity, reading acuity, and critical print size, pre and post training

	Pre training	Post training	*p* value
N (M/F)	33 (15/18)	33 (15/18)	-
Visual acuity (logMAR)			
• Binocular	0.64 ± 0.22	0.59 ± 0.23	< 0.001
• Better eye	0.59 ± 0.25	0.58 ± 0.25	0.008
• Binocular ratio	0.91 ± 0.13	0.95 ± 0.13	0.02
Contrast sensitivity (logCS)			
• Binocular	1.00 ± 0.31	1.14 ± 0.31	< 0.001
• Better eye	1.02 ± 0.29	1.11 ± 0.29	0.002
• Binocular ratio	0.99 ± 0.15	1.03 ± 0.14	0.07
Reading acuity (logMAR)			
• Binocular	0.87 ± 0.27	0.79 ± 0.28	< 0.001
• Better eye	0.78 ± 0.30	0.74 ± 0.31	< 0.001
• Binocular ratio	0.90 ± 0.17	0.93 ± 0.17	0.007
Critical print size (logMAR)			
• Binocular	1.09 ± 0.30	0.96 ± 0.33	< 0.001
• Better eye	0.95 ± 0.36	0.90 ± 0.34	0.004
• Binocular ratio	0.85 ± 0.17	0.93 ± 0.13	< 0.001

### Effect of PRL correspondence on binocular ratios of reading parameters post training

Post training, 24 out of 33 patients had the monocular PRLs in corresponding locations in the two eyes, while 9 patients did not. We examined whether fixation stability and BRs of reading parameters differed for the two subgroups. Independent-sample t-tests revealed that pre training, there was no significant difference in any of these outcome measures between the two subgroups. Post training, fixation stability of the better eye and of the worse eye did not differ significantly for the two subgroups. However, BR of the reading parameters were marginally higher in the PRL correspondence subgroup than in the PRL no correspondence subgroup (*p* values from 0.04 to 0.06), suggesting that the PRL correspondence had a positive impact on binocular function. The results are presented in Table 2.

**Table 2:** Mean (± standard deviation) of the outcome measures for the subgroup with the PRLs in corresponding locations and for the subgroup with the PRLs in non-corresponding locations post training. Independent-sample t-tests *p* values are shown in the last column

	PRL correspondence *N* = 24	PRL no correspondence *N* = 9	*p*
Fixation stability (deg^2^)			
• Better eye	1.85 ± 1.21	1.75 ± 1.13	0.8
• Worse eye	3.51 ± 2.14	3.17 ± 1.27	0.7
Binocular ratio			
• Max reading speed	0.91 ± 0.09	0.84 ± 0.12	0.06
• Critical print size	0.95 ± 0.12	0.85 ± 0.13	0.04
• Reading acuity	0.96 ± 0.14	0.84 ± 0.21	0.06

## Discussion

This study examined whether a new method of BF training aimed at improving fixational control and at bringing the monocular PRLs into correspondence on functioning retina in both eyes (if needed) could improve binocular function of patients with binocular reading inhibition due to central vision loss. There are several important findings of this study. First, we found that BF training led to better fixation stability in both eyes and – for cases that allowed it – to retinal correspondence of the monocular PRLs. Second, BF training resulted in a significant increase in binocular reading speed and in a relief of binocular reading inhibition. Third, this rehabilitation method led to an enhancement of other binocular functions along with better binocular summation on these measures. Finally, having monocular PRL correspondence on functioning retina in the two eyes post training facilitated the improvements in binocular function.

We found that BF training resulted in better fixational control in both eyes, only after 10 ten-min long sessions for each eye. Fixation stability improved remarkably both in the better and in the worse eyes of the patients included in this study. Moreover, PRL correspondence was achieved in an important proportion of patients. Generally, BF training targets fixation stability and PRL relocation, and its efficiency has been demonstrated repeatedly [16–19]. However, due to technological limitations, BF training is possible only for monocular, but not for binocular viewing. Typically, only the better eye is trained, and it has been shown that the gain in fixation stability after BF training of this eye is related to better monocular visual functions [16, 19]. We are not aware of any studies using the BF training for both eyes to improve binocular function and to ameliorate binocular inhibition, even though binocular viewing is the more naturalistic viewing condition. However, the important question is, do better fixational control and PRL correspondence achieved through BF training in both eyes translate into improved binocular performance in patients with reading inhibition?

The answer to this question is a clear yes. All patients included in this study initially had a reading speed that was worse during binocular viewing than during monocular viewing with the better eye. Training resulted in a significant improvement in binocular reading speed, from an average of 57 wpm pre training to 67 wpm post training, but interestingly, the gain in the monocular reading speed was modest. However, binocular reading inhibition ameliorated considerably from an average BR of 0.78 pre training to 0.89 post training, with 30% of patients attaining a complete reversal of binocular inhibition (i.e., BR > 0.95). This represents an encouraging finding, given that binocular inhibition adds to the already devastating list of visual impairments produced by macular disease.

Among those whose binocular inhibition was resolved, only 2 patients maintained the monocular PRLs in non-corresponding locations post training. This highlights the advantage of PRL correspondence on functioning retina in both eyes for binocular function in patients with central scotomas in both eyes. However, this may not always be achievable or even indicated during rehabilitation process for all cases [9]. For example, in patients with a PRL in close proximity to the former fovea in the better eye and a PRL in far eccentricity in the worse eye, a PRL correspondence on functioning retina would be achieved only through a relocation of the PRL in the better eye also in far eccentricity. Evidently, this is not desirable since visual acuity decreases sharply with eccentricity, and also may not be possible since the visual system will always prefer a PRL location closer to the fovea, if available [9]. In addition, for patients with relatively equal central lesions, the monocular PRLs are often in corresponding retinal locations, but these locations sometimes are in the nasal/temporal retina, known for providing a reduced visual span that severely impairs reading performance [17–19, 27]. In this case, if a PRL relocation superior or inferior to the central lesion is considered for the better eye in order to provide a wider visual span, then it is paramount to check the retinal correspondence in the worse eye such as the PRL in this eye would fall on functioning retina during binocular viewing. Otherwise, it is possible that the PRL in the worse eye would shift from a functioning to a non-functioning retina after the PRL relocation in the better eye, and this would affect binocular function. Therefore, care should be taken when selecting the patients for vision rehabilitation aimed at relocating the PRL, particularly in the better eye.

We also examined whether the improvements due to BF training were specific to reading speed or they transferred to other binocular functions. Patients included in this study were selected to have binocular inhibition of reading speed. There are particular requirements for optimal reading performance which are not necessarily needed for other visual functions in patients with central vision loss. For example, a PRL located in the proximity of the former fovea and to the left of the scotoma in the visual field would provide a reduced visual span with catastrophic consequences for reading speed [20, 28], but visual acuity may not be seriously affected. Indeed, we found that BF training resulted in statistically significant improvements in binocular and monocular visual acuity and contrast sensitivity, but the clinical relevance of these improvements was minor. However, an important gain was obtained for the critical print size, both in binocular performance and in BR.

Post training, less than a third of the patients did not achieve PRL correspondence in the 2 eyes and their binocular ratios of reading parameters were worse (although statistically marginal) than of those with corresponding monocular PRLs. This suggests that achieving monocular PRL correspondence on functioning retina is also an important factor for improving binocular function. More research involving eye tracking during binocular and monocular viewing would be needed to shed more light into these findings. Moreover, future research should examine the long-term effect of the biofeedback intervention presented in this study.

In conclusion, this study examined the effect of a new approach of using BF training in patients with binocular reading inhibition due to central vision loss. We found that this method was effective in alleviating binocular reading inhibition. The results add much needed value to the field, given the lack of data in the literature addressing the issue of rehabilitation for patients with binocular inhibition due to central vision loss. Since binocular inhibition represents another layer of visual impairment to already devastating vision loss due to macular diseases, we recommend that this rehabilitation method should be considered for better management of these patients in order to achieve successful functioning results.

## Abbreviations

*BCVA*: Best corrected visual acuity
*BCEA*: Bivariate contour ellipse area
*BF*: Biofeedback
*BR*: Binocular ratio
*CS*: Contrast sensitivity
*Deg*: Degree
*ETDRS*: Early Treatment of Diabetic Treatment Study
*Hz*: Hertz
*IAPB*: International Agency for the Prevention of Blindness
*MP*: Microperimeter
*PRLs*: Preferred retinal loci
*PRL*: Preferred retinal locus
*SE*: Standard Error
